# Risk factors for the presence of dengue vector mosquitoes, and determinants of their prevalence and larval site selection in Dhaka, Bangladesh

**DOI:** 10.1371/journal.pone.0199457

**Published:** 2018-06-21

**Authors:** Kishor Kumar Paul, Parnali Dhar-Chowdhury, C. Emdad Haque, Hasan Mohammad Al-Amin, Doli Rani Goswami, Mohammad Abdullah Heel Kafi, Michael A. Drebot, L. Robbin Lindsay, Gias Uddin Ahsan, W. Abdullah Brooks

**Affiliations:** 1 Program for Emerging Infections, Infectious Diseases Division, International Centre for Diarrhoeal Disease Research, Bangladesh (icddr,b), Dhaka, Bangladesh; 2 Natural Resources Institute, University of Manitoba, Winnipeg, Manitoba, Canada; 3 National Microbiology Laboratory, Public Health Agency of Canada, Winnipeg, Manitoba, Canada; 4 Department of Public Health, North South University, Dhaka, Bangladesh; 5 Department of International Health, Johns Hopkins Bloomberg School of Public Health, Baltimore, Maryland, United States of America; Swedish University of Agricultural Sciences, SWEDEN

## Abstract

Dengue viruses are responsible for over 100 million infections a year worldwide and are a public health concern in Bangladesh. Although risk of transmission is high, data on vector population characteristics are scanty in Bangladesh; therefore, a comprehensive prediction of the patterns of local virus transmission is not possible. Recognizing these gaps, multi-year entomological surveys were carried out in Dhaka, where the disease is most frequently reported. The specific objectives of the present study are threefold: i) to determine the risk factors for the presence of *Aedes* mosquitoes; ii) to identify the types of most productive and key containers; and iii) to estimate the effects of climatic factors on *Aedes* abundance in the city of Dhaka, Bangladesh. Entomological surveys were conducted in 12 out of 90 wards in Dhaka. These wards were selected using a probability proportional sampling procedure during the monsoon seasons in 2011, 2012 and 2013 and in the dry season in 2012. All containers inside and around sampled households were inspected for mosquito larvae and pupae, and containers were classified according to their relative size, use pattern, and materials of construction. During the study period (2011–2013), 12,680 larvae and pupae were collected. About 82% of the identified immature mosquitoes were *Aedes aegypti*, while the remainder were *Ae*. *albopictus* and other mosquito species. The largest number of immature mosquitoes was collected from tires and refrigerator trays during 2011 and 2012 monsoon seasons. Conversely, plastic drums were the most productive during the 2012 dry and 2013 monsoon season. Vehicle parts and discarded construction materials were the most efficient producers of *Aedes* mosquitoes in all surveys. The presence of *Aedes* mosquitoes was significantly (p < 0.05) higher in low socio-economic zones of Dhaka. Container location, presence of vegetation, and availability of shade for containers were also significantly associated with finding immature *Aedes* mosquitoes, based on multivariable analysis after confounder adjustment. Rainfall, temperature, and relative humidity also significantly affected the mean abundance of mosquitoes. Proper use, disposal, and recycling of the containers that effectively produce large numbers of *Aedes* vector mosquitoes may decrease the risk of arboviral transmission.

## Introduction

Dengue fever is a self-limiting illness and the four dengue virus serotypes are the most frequent global arboviral infection of humans. Over 3.9 billion people from 128 countries live under the threat of dengue and 390 million infections occur every year, with 96 million people experiencing clinical manifestations of dengue [1,2]. In Bangladesh, the first dengue cases were detected in 1964 and the disease was referred to as ‘Dacca fever’ [3]. From 1964 to 1999, sporadic cases of dengue were reported but it was not considered as a major public health concern. However, in 1999 a major outbreak took place the capital city, Dhaka, followed in 2000 by a countrywide epidemic involving 5,551 cases and 93 deaths [4–7]. In addition to dengue, chikungunya fever outbreaks, another *Aedes*-borne viral disease, was reported in two different geographical regions of Bangladesh in 2008 and 2011 [8,9]. In 2016, with the help of World Health Organization, Zika virus infection was detected for the first time in Bangladesh [10].

Further outbreaks of dengue were reported from 2000 to 2009 and more than 90% of all of the dengue cases were reported from Dhaka, and hence, this capital city was identified as the most endemic urban area for dengue in the country [7]. Further to this, a serosurvey conducted during 2012 in 12 administrative wards of Dhaka observed dengue antibodies among more than 80% of the study participants [11].

*Aedes* (*Stegomyia*) *aegypti* Linnaeus and *Aedes* (*Stegomyia*) *albopictus* Skuse are the known vectors of dengue virus and other arboviruses including chikungunya and zika virus [12]. These species are highly efficient vectors of arboviruses and live in close proximity to humans [13]. Both of these species were first recorded in Bangladesh during 1952 [14] and studies conducted in 1980 showed high abundance of both of these species in Dhaka [15]. In 1997, a Breteau Index (BI) of 30.8 was recorded which was well above the risk levels for dengue transmission [16]. During the 2000 dengue outbreak a comprehensive entomological survey was conducted in all wards of Dhaka, and an equally high BI of 24.6 was reported [17]. *Aedes aegypti* and *Ae*. *albopictus* mosquitoes lay their eggs and larvae subsequently develop in domestic water storing containers, rainwater-holding objects including a wide variety of discarded materials as well as natural water retaining structures like tree holes and plant axils that are often abundant in peri-domestic environments. Thus there is a wide range of potential larval development sites for these mosquito species that vary in size, shape and location in the environment[17]. Though many different types of containers can serve as development sites for vector mosquitoes, in some cases selected containers produce large numbers of larvae/pupae or some containers are sufficiently abundant to be efficient sources of larvae/pupae. Thus, these “key containers” are either highly productive or highly efficient, and both drive the local abundance of vector mosquitoes and are the targets for source reduction so important to vector suppression and dengue prevention. Vector abundance varies seasonally because of local changes in temperature, humidity and rainfall that affect the availability of larval development sites [18]. In addition, the larval development sites for *Aedes* mosquitoes also vary spatially [19]. Mosquitoes may concentrate in some parts of urban centres and display local scale variations within an urban centre [20]. As a result, a clear and comprehensive understanding of the spatial distribution of key containers and how these can change on a seasonal basis is essential for designing effective vector control programs. Notably, data on *Aedes* abundance, and how it changes over the seasons in Dhaka are scanty.

International Centre for Diarrhoeal Disease Research, Bangladesh (icddr,b) in collaboration with the University of Manitoba, Canada, the Public Health Agency of Canada, North South University, Bangladesh, and Directorate General of Health Services under Ministry of Health and Family Welfare of the Government of Bangladesh, pursued the present study to understand the dynamics of dengue vectors in Dhaka by conducting entomological surveys for *Aedes* mosquitoes. The results of the first survey, conducted during the monsoon season in 2011, were published elsewhere [21]. In this study, we have combined the outcomes of four entomological surveys conducted in 2011, 2012 and 2013 in order to examine seasonal patterns in the spatial and temporal abundance of the immature stages (larvae and pupae) of *Aedes* mosquitoes, to identify the most productive and efficient container types for these species and to determine some of the factors affecting the abundance of *Aedes* larvae in Dhaka. The specific objectives of the present study are threefold: i) to determine the risk factors for the presence of *Aedes* mosquitoes; ii) to identify the types of most productive and key containers; and iii) to estimate the effects of climatic factors on *Aedes* abundance in the city of Dhaka, Bangladesh.

## Methods

### Study period

Three household based entomological surveys were conducted in 12 of 90 wards in Dhaka [20] at times when dengue incidence was anticipated to be high [7] and included the monsoon periods of 2011 (July-August), 2012 (July) and 2013 (August-September). In addition, a pre-monsoon (dry season) entomological survey was carried out in 2012 (March) when dengue incidence was assumed to be low.

### Study area

**Fig 1:**
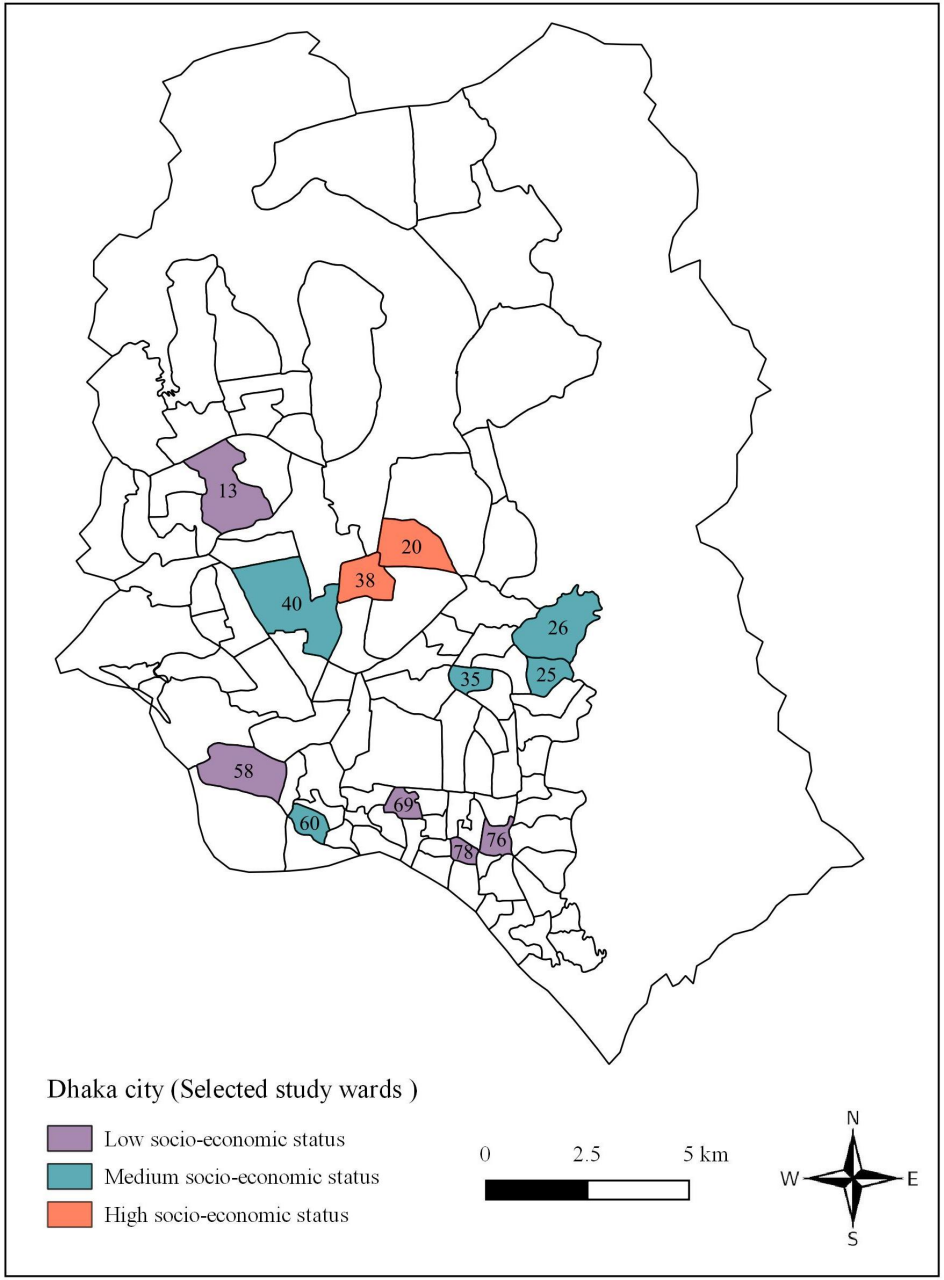
Location of the study areas (12 selected city wards) within Dhaka, Bangladesh.

Dhaka, the capital of Bangladesh, is one of the most densely populated cities in the world with more than 12 million people living in an area of about 360 km^2^, and it is divided into 90 local administrative units—called ‘wards’. The city usually experiences a hot, wet, and humid tropical climate, with monthly mean temperature varying between 20°C (68°F) in January and 32°C (90°F) in May. Using the Delphi method [22], the administrative wards were categorized into three socioeconomic status zones (SESZ), which produced 36 wards designated as low SESZ (LSESZ), 40 wards as medium SESZ (MSESZ), and 14 wards as high SESZ (HSESZ) (Fig 1). The indicators used for SESZ categorization included: a) municipal property tax rates, b) property market value, c) rate of property rent, d) proximity of types of markets and shopping areas, e) types of building structure, f) proximity to public services, g) state of infrastructure, and h) state of transport in each ward. With respect to cost, human resource requirements, and the time needed, as well as to minimize the margin of error in making population inferences, we determined that 12 wards were adequate for representative entomological surveys for the entire city. By employing a probability proportional to the number of wards in SESZs sampling method, a total of 12 administrative wards (2 wards from HSESZ, 5 wards from MSESZ, and 5 wards from LSESZ) were selected. Because the primary sampling unit was a household (HH), a random sample of 100 HHs were targeted from each ward, resulting in 1200 HHs as sampling units for each survey. For HH surveys, a spatial randomization procedure was followed by drawing grid cells on a map of each ward and a total of 100 HHs were selected using a random number table as described by Dhar-Chowdhury et al [21]. The survey was repeated, where possible, in the same HHs for each of the subsequent three surveys.

### Entomological collections

All selected sites were physically marked on the map and their addresses were recorded on both field data sheets and GPS units. All containers inside the HHs as well as within a 50-meter radius of the HHs were inspected for immature mosquitoes. Any containers that held water for more than 3 days were considered “wet containers” [23]. The amount of water in these containers at the time of the survey was recorded, and respondents were asked about the source and use of water within each container type. Water within any outdoor containers, without any nearby water source, during the monsoon season was considered “rainwater”. The number and type of “dry containers” (ones holding water for less than 3 days) were also recorded in order to calculate the container index (CI). All wet containers were examined for *Aedes* larvae and/or pupae. Only single storied or ground floor apartments were inspected because of the lack of access to the upper floors and roofs of the buildings and any containers on rooftops such as rooftop water tanks, flower tubs and other discarded materials were not inspected. Collection of larvae and pupae and the identification procedures were described elsewhere [21]. Species determinations were performed only on a subset of the immature mosquitoes collected during each survey but the sub-sampling was done randomly and the subset of data is representative of the species composition across all households and years when the surveys were conducted.

### Classification of containers used as larval development sites

All wet containers were categorized into seven classes depending on their use in daily life, manufacture materials, and their relative size or volume. Class 1 containers were comprised of small (i.e., 1 ml to 1 l of water) plastic containers that are used for domestic purposes (e.g., water storage). Class 2 containers were discarded but recyclable vehicle parts and construction materials. Class 3 containers consisted of medium (i.e., >1 and < 5 l) to large (>5 l) water reservoirs that are used regularly for water storage. Class 4 containers included small to medium sized non-plastic containers, most of which were used for temporary water storage. Class 5 containers were discarded HH materials. Class 6 containers consisted of ornamental containers typically used to capture water run-off from plants. Class 7 containers included natural plant based materials such as coconut shells, plant axils and tree holes. Immature *Aedes* mosquitoes were detected in these classes of containers during the course of this study.

### Collection of meteorological data

Meteorological data including rainfall, temperature and relative humidity for monsoon (June-September) and pre-monsoon (February-March) from 2011 to 2013 were collected from Bangladesh Meteorological Department, Dhaka, Bangladesh. Such temporal rainfall, temperature and relative humidity daily data coverage ensured corroboration of meteorological data for previous 30 days of the entomological survey periods (2011: July-August; 2012: March; July; 2013: August-September) enabling to assess lag time effects of climatic factors on vector abundance.

### Statistical analysis

*Stegomyia* indices including house index (HI), the percentage of houses infested with larvae and/or pupae; container index (CI), the percentage of positive containers; BI, the number of positive containers per 100 HHs and pupal index (PI), number of pupae per 100 houses inspected [15] and their 95% confidence intervals (CI) were calculated using the exact binomial test. Descriptive analysis was conducted for the distribution of wet containers and *Aedes* immatures for the four surveys. Wet containers with any number of larvae or pupae were considered “positive containers” (PC). Houses with PCs were considered “positive premises”. Following Tun-Lin et al.’s study [24], we considered “Key premises” (KP) as those contained ≥3 PCs either indoors or outdoors. The contribution of different types of containers on the production of *Aedes* mosquitoes was analyzed by calculating the percentages of positive containers, mean immatures with 95% confidence interval for each category of container, container productivity (number of immatures/all immatures X 100), and container efficiency (productivity/prevalence of container) [25]. A container efficiency of 1.0 was considered when all containers were assumed equally efficient [26].

Prevalence of container was calculated by dividing the number of wet containers with all containers. We performed the univariate logistic regression method for each *Aedes* species to calculate odds ratio to identify associations between individual factors and the presence of larvae and/or pupae. As explanatory factors, we considered six variables including socio-economic status of the HHs, type or source of water, location of container (in-or out-doors), presence of vegetation near containers, and the amount of shade for the containers. Following these procedures, we constructed a conceptual framework to identify factors of interest for the final multivariable logistic regression model. A generalized linear model was used to investigate the association between climatic variables and the abundance of *Aedes* immatures. In the analysis, the abundance of mosquitoes per container was considered as the outcome variable and mean temperature, total rainfall, and percent relative humidity of the preceding month were used as predictor variables to make possible adjustments. All data collected were analyzed using the statistical software package STATA 13.1 (StataCorp LP, TX, USA).

### Ethics approval and consent to participate

This study was approved by the Bangladesh Medical Research Council, icddr,b research and ethical review committees, and the Joint Faculty Research Ethics Board at the University of Manitoba, Canada. The purpose and objectives of the study, benefits and risks, and their voluntary participation option were explained to the head of each HH. As culturally appropriate, such informed-consent procurement sessions were witnessed by the neighbours and close kin to validate and witness verbal consent. After obtaining informed oral consent, the HH premises were inspected for the presence of containers and discarded materials that can store water and immature stages of mosquitoes.

## Results

The annual response rate was 74%, 60% and 53% in 2011, 2012 and 2013 respectively, almost equally distributed among SESZs, and thus majority of the respondents volunteered to participate in the study each year. A total of 884 HHs participated in the first survey (2011). Depending on the availability of survey time, alternative HHs were selected if the residents of the randomly selected HH refused to participate or were not at home during survey. In dry months, dengue incidence usually becomes very low in Bangladesh [7]. This influenced the second survey (2012 dry season) as many previous participants refused to continue to participate. We observed a large number of people who participated in the previous survey were not available in subsequent surveys as moving residential location of HHs within Dhaka in a short period of time is frequent, attributed to the fact that most of the city dwellers live in rented apartments. As a result, members of only 546 HHs consented in the second survey (2012 dry season). The third (2012 monsoon season; n = 899) and fourth (2013 monsoon season; n = 639) surveys were conducted during the monsoon months and the number of respondents were higher, primarily due to restricted residential shifts during monsoon seasons (Table 1).

**Table 1:** Statistics on sites used by *Aedes* mosquitoes and *Stegomyia* indices generated during larval survey conducted from 2011 to 2013 in Dhaka, Bangladesh.

Coverage, Outcomes and Indices	Survey period			
	2011 Monsoon	2012 Dry	2012 Monsoon	2013 Monsoon
Houses inspected	884	546	899	639
Wet containers inspected	1,260	463	689	592
No. indoor positive containers	147	32	96	51
No. outdoor positive containers	346	21	161	93
Total positive containers (Percent positive)	493 (39)	53 (11)	257 (37)	144 (24)
Immature *Aedes* number	4,217	514	5,554	2,395
HI (95% CI)	25.0 (22.2–28.0)	7.1 (5.3–9.6)	24.7 (22.0–27.6)	16.6 (14.0–20.0)
CI (95% CI)	39.0 (36.4–41.8)	11.4 (8.8–14.7)	37.4 (33.6–40.8)	24.3 (21.0–27.9)
BI (95% CI)	55.8 (47.0–64.5)	9.7 (6.5–13.0)	28.7 (24.6–32.9)	22.5 (18.0–27.0)
PI (95% CI)	62.2 (44.2–80.2)	16.7 (6.3–27.0)	153.5 (91.4–215.6)	75.9 (50.5–101.2)

### Numbers of water-holding containers inspected

Our entomological survey results revealed that that the proportion of positive containers has been decreasing gradually over the successive rounds of sampling, from a high of 39% in 2011 to only 24% in 2013 (Table 1). Such a trend was reflected in the consistently declining gradient from 2011 to 2013, with an expected low proportion during the dry season (11% during 2012 pre-monsoon).

### Species determination of collected *Aedes* immatures

A total of 12,680 *Aedes* larvae and pupae as well as 268 larvae of other mosquitoes were collected during the four surveys. Highest (n = 5,554) number of immatures were collected during the monsoon season of 2012 and lowest number (n = 514) were collected during dry season of 2012 (Table 1). Species determination of 5,078 (39%) immature mosquitoes was carried out, of which 82% (4,152) immatures were identified as *Ae*. *aegypti*, 13% (658) were *Ae*. *albopictus*, and 5% (268) were of other mosquito species including *Culex quinquefasciatus* and *Armigeres subalbatus*. The overall proportion of *Ae*. *aegypti* ranged from 72% to 85%, and was highest during the 2011 monsoon and lowest during the 2012 monsoon. In contrast, the overall proportion of *Ae*. *albopictus* ranged from 8% to 23%, and was highest during the 2012 monsoon and lowest during the 2013 monsoon season.

### *Stegomyia* indices according to seasons and SESZ

The HI, CI and BI were found to be lower during 2013 monsoon compared to 2011 monsoon season (Table 1). However, PI was higher in 2012 than in 2011. Noticeably, the *Stegomyia* indices were highest in wards located in LSESZ and were lowest in wards located in MSESZ (Table 2). The PI did not show any successive pattern; it was highest in HSESZ relative to two other zones.

**Table 2:** Statistics on sites used by *Aedes* mosquitoes and *Stegomyia* indices generated within three socio-economic zones surveyed from 2011 to 2013 in Dhaka, Bangladesh.

Coverage, Outcomes and Indices	Socio-Economic Status Zones		
	Low	Medium	High
Houses inspected	909	910	418
Wet containers inspected	1,182	1,184	638
No. indoor positive containers	158	113	55
No. outdoor positive containers	264	244	111
Total positive containers (Percent positive)	422 (36)	357 (30)	166 (26)
Immature *Aedes* number	5,047	5,078	2,555
HI (95% CI)	25.6 (22.9–28.6)	22.0 (19.4–24.8)	25.1 (21.2–29.5)
CI (95% CI)	35.6 (32.9–38.4)	30.1 (27.5–32.7)	26.3 (23.1–29.9)
BI (95% CI)	46.4 (39.4–53.4)	39.3 (32.3–46.4)	40.2 (30.9–49.5)
PI (95% CI)	111.1 (81.0–141.2)	109.7 (54.7–164.6)	119.1 (62.8–175.5)

### Positive and key premises for vector production over 2011–2013

It is important here to analyse and reiterate positive and key premises to provide the context of container analysis. During the three consecutive monsoon seasons of 2011, 2012 and 2013, 25% (220 of 884), 24% (220 of 899), and 17% (106 of 639) of the inspected houses were positive for *Aedes* larvae and/or pupae, respectively. During 2012 dry season, only 7% (39 of 546) of the inspected houses were observed to be positive for *Aedes* (S1 Table). Percentage of KP decreased from 37% in 2011 monsoon to 10% in 2012 monsoon; however, it increased to 22% during the 2013 monsoon season (S1 Table).

### Main types of positive containers and most productive containers by season

Types of Positive Containers (PCs) varied from 20 to 32 during all four surveys. In each sampling period, only 10 different types of containers produced about 80% of the *Aedes* immatures. Tires, usually discarded (in open place), produced highest number of immatures during 2011 monsoon (11.3%) and 2012 monsoon seasons (12.5%) while plastic drums, which are used to store water indoors, yielded most *Aedes* during 2012 dry season (28.2%) and 2013 monsoon seasons (17.6%). Overall, tires, flower tub and tray, refrigerator tray, and plastic drum (sealable) produced higher proportion of *Aedes* immatures compared to other containers (Table 3).

**Table 3:** Distribution of most productive containers infested with *Aedes* larvae and/or pupae in households inspected in Dhaka, Bangladesh, 2011–2013.

Container types	2011 Monsoon (Wet)		2012 Pre-monsoon (Dry)		2012 Monsoon (Wet)		2013 Monsoon (Wet)		Cumulative percentage
	N	%	N	%	N	%	N	%	%
Refrigerator tray	396	9	0	---	630	11	397	17	11.3
Tires	474	11	9	2	690	13	185	8	22.0
Plastic drum (sealable)	389	9	145	28	392	7	421	18	32.7
Flower tub & tray	470	11	30	6	373	7	315	13	42.1
Plastic bucket	366	9	41	8	439	8	203	8	50.4
Water Tank	186	4	40	8	686	12	102	4	58.5
Clay pot	338	8	22	4	360	7	254	11	66.2
Disposable Plastic Containers	335	8	0	---	151	3	3	0	70.0
Plastic bottle	329	8	1	0	81	1	35	1	73.6
Flooded floor	0	---	13	3	320	6	95	4	77.0
Earthen jar (Motka)	34	1	23	5	201	4	61	3	79.5
Money plants tub	142	3	85	17	41	1	28	1	81.8
Tree leaves	0	---	0	---	270	4	4	0	84.0
Plastic sheet to cover large object	66	2	0	---	57	1	33	1	85.2
Plastic bags	36	1	24	5	12	0	0	---	85.8
Plant axil	26	1	30	6	2	0	3	0	86.3
Glass bottle	9	0	20	4	0	---	0	---	86.5
Other productive containers (20 other types)	617	15	31	6	796	14	256	11	100
Total (all containers)	4,213	100	514	100	5,501	100	2,395	100	---

N = No. of immature *Aedes*

### Determining the key containers

Considering immature production in all four survey periods, the class 1 containers were most abundant in the inspected HHs and their surroundings. These were the most productive (29.4) but least efficient (0.6) containers among all the container classes. Although class 2 containers were neither most abundant nor most productive, they were the most efficient containers (3.3) to produce immature *Aedes*. Class 1 and class 2 containers can therefore be distinguished as the key containers for *Aedes* immature production in Dhaka. The remaining classes of containers were moderately abundant and efficient (Table 4).

**Table 4:** Role of different classes of containers in the production of *Aedes* mosquitoes in Dhaka, Bangladesh, 2011–2013.

Container class	Container types^a^	Containers	Percentage PCs	Mean immatures (95% CI)	CP	CE
1	Small plastic reservoirs	Bottle, bucket, bag, mug, and small drum	35.5	11 (10,13)	29.4	0.6
2	Vehicle and construction discards	Tires, battery shell, wood slab, and cement mixer	6.8	22 (17,26)	10.9	3.3
3	Medium-Large water reservoirs	Water tank and metal drum	10.5	17(13,22)	13.5	1.3
4	Small to medium non-plastic reservoirs	Clay pot, aluminum pot, glass bottle, tin/metal can, and metal bucket	15.5	12(10,14)	13.6	1.1
5	Discarded HH materials	Refrigerator tray, tarp to cover large objects, broken toilet parts, and musical instruments	15.1	14(12,17)	16.5	1.3
6	Flower tubs	Flower tub, flower tray, and money plant (*Epipremnum aureum*) tub	13.2	12(10,14)	11.8	1.3
7	Plant materials	Dry or green coconut shell, plant axil, tree holes, and bamboo stamp	3.5	17(8,25)	4.4	1.2

^a^The volume of water held by containers is as follows: small (1 ml to 1 l), medium (>1 and < 5 l) and large (> 5 l).

PC = Positive containers

CP = Container productivity (no. of immatures x 100/all immatures)

CE = Container efficiency (productivity/prevalence of container), prevalence of container = no. of wet containers x 100/all containers

### Risk factors for the presence of *Ae*. *aegypti*

From bivariate analysis, it was observed that the SESZ, type of water, location of container, presence of vegetation and shade was associated with presence of *Ae*. *aegypti* immatures. In the multivariable analysis after adjusting for possible confounders, the presence of *Ae*. *aegypti* was found to be significantly high in the low SESZ area (OR 1.69, CI: 1.17–2.44) and in containers holding rain water (OR 1.92, 95% CI: 1.35–2.72). Containers located outdoors (OR 1.92, 95% CI 1.48–2.50) and wet containers which were proximal to vegetation (OR 1.50, 95% CI: 1.10–2.04) were more likely to have *Ae*. *aegypti*. More larvae and pupae were detected in containers without shade compared to the containers located in areas with full shade (OR 2.30, 95% CI 1.55–3.43) (Table 5).

**Table 5:** Risk factors for wet containers to harbour *Aedes aegypti* larvae in Dhaka, Bangladesh, 2011–2013.

Predictor variables	Wet containers	Positive (%)	Bivariate analysis OR (95% CI)	*p—value*	Multivariable analysis Adjusted OR (95% CI)	*p—value*
Socio-Economic Status Zone^a^						
High	635	9.0	1		1	
Medium	1,179	11.2	1.27 (0.88–1.86)	0.198	1.23 (0.85–1.80)	0.265
Low	1,179	14.8	1.75 (1.23–2.52)	0.002	1.69 (1.17–2.44)	0.005
Type of water^b^						
Tap	1,682	9.3	1		1	
Rain	1,041	18.4	2.19 (1.69–2.85)	≤0.001	1.92 (1.35–2.72)	≤0.001
Tube well	53	11.3	1.25 (0.46–3.39)	0.660	1.21 (0.45–3.27)	0.702
Ring well	14	7.1	0.75 (0.10–5.83)	0.787	0.68 (0.09–5.28)	0.716
Others*	183	4.4	0.45 (0.22–0.93)	0.031	0.46 (0.22–0.97)	0.042
Container location^c^						
Indoor	1,548	8.7	1		1	
Outdoor	1,425	16.0	1.98 (1.53–2.56)	≤0.001	1.92 (1.48–2.50)	≤0.001
Vegetation^d^						
None	2,089	10.2	1		1	
Nearby	577	18.0	1.92 (1.47–2.51)	≤0.001	1.50 (1.10–2.04)	0.010
Under	296	15.2	1.56 (1.04–2.34)	0.033	1.31 (0.84–2.06)	0.237
Shade^e^						
None	1,289	12.8	1.71 (1.28–2.28)	≤0.001	1.31 (0.91–1.89)	0.148
Partial	527	20.1	2.93 (2.08–4.14)	≤0.001	2.30 (1.55–3.43)	≤0.001
Full	1,130	7.9	1			

OR: Odds ratio

*Others include any undetermined source of water

^a^ adjusted for Vegetation, Container location, Shade and Container class

^b^ adjusted for Container location

^c^ adjusted for Socio-Economic Status Zone

^d^ adjusted for Container location, Socio-Economic Status Zone

^e^ adjusted for Container location, Socio-Economic Status Zone

### Risk factors for the presence of *Ae*. *albopictus* are similar to *Ae aegypti*

From the multivariable analysis after adjustment for possible confounders, the presence of *Ae*. *albopictus* was observed to be significantly high in containers with rainwater (OR 2.98, 95% CI 1.09–8.13). Containers located outdoors were also more likely to harbour *Ae*. *albopictus* than containers located indoors (OR 5.05, 95% CI 2.33–10.96). Detection of *Ae*. *albopictus* was significantly more likely when the container was in close proximity to vegetation (for container under vegetation—OR 3.94, 95% CI 1.70–9.16; for container located nearby vegetation—OR 3.12, 95% CI 1.45–6.68). The containers with no shade were also significantly more likely to harbour immature *Ae*. *albopictus* (OR 3.96, 95% CI 1.33–11.82) (Table 6).

**Table 6:** Risk factors for wet containers to harbour *Aedes albopictus* larvae in Dhaka, Bangladesh, 2011–2013.

Predictor variables	Wet containers	Positive (%)	Bivariate analysis OR (95% CI)	*p—value*	Multivariable analysis Adjusted OR (95% CI)	*p—value*
Socio-Economic Status Zone^a^						
High	635	1.9	1		1	
Medium	1,179	2.3	0.75 (0.34–1.72)	0.510	0.61 (0.26–1.42)	0.250
Low	1,179	1.4	1.22 (0.58–2.55)	0.597	0.99 (0.47–2.07)	0.973
Type of water^b^						
Tap	1,682	0.8	1		1	
Rain	1,041	3.9	5.23 (2.70–10.13)	≤0.001	2.98 (1.09–8.13)	0.033
Tube well	53	0				
Ring well	14	0				
Others*	183	1.09	1.42 (0.32–6.36)	0.646	1.74 (0.35–8.77)	0.501
Container location^c^						
Indoor	1,548	0.7	1		1	
Outdoor	1,425	3.2	5.10 (2.38–10.91)	≤0.001	5.05 (2.33–10.96)	≤0.001
Vegetation^d^						
None	2,089	0.9	1		1	
Nearby	577	4.2	4.98 (2.58–9.59)	≤0.001	3.12 (1.45–6.68)	0.003
Under	296	4.7	5.67 (2.59–12.37)	≤0.001	3.94 (1.70–9.16)	0.001
Shade^e^						
None	1,289	3.1	7.17 (2.76–18.61)	≤0.001	3.96 (1.33–11.82)	0.014
Partial	527	1.9	4.34 (1.43–13.20)	≤0.001	2.37 (0.54–10.31)	0.248
Full	1,130	0.4	1			

*Others include any undetermined source of water

^a^ adjusted for Vegetation, Container location, Shade and Container class

^b^ adjusted for Container location

^c^ adjusted for Socio-Economic Status Zone

^d^ adjusted for Container location, Socio-Economic Status Zone

^e^ adjusted for Container location, Socio-Economic Status Zone

### Effect of climatic factors on the abundance of *Aedes* mosquitoes

**Fig 2:**
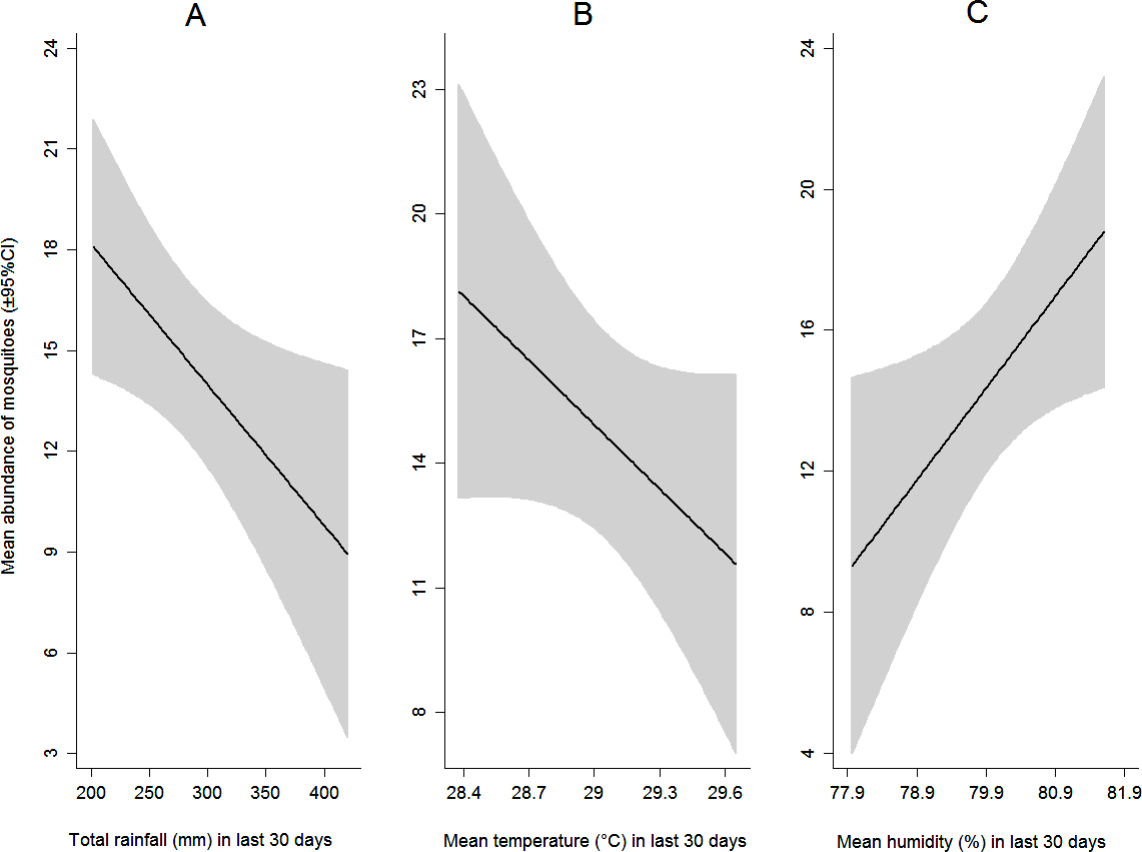
Effects of climate variables on the abundance of larvae and/or pupae of *Aedes* mosquito during monsoon (wet) season. Panels A, B and C show the effects of total rainfall, mean temperature (°C), and mean relative humidity (%) during last 30 days of survey, on the abundance of *Aedes* per container.

The mean abundance of immature *Aedes* was significantly influenced by the selected climatic variables. Total rainfall (Fig 2A) and mean temperature (Fig 2B) in previous 30 days of entomological sampling had a significant negative effect on *Aedes* abundance. Each mm increase in total rainfall decreased the mean number of immature *Aede*s per positive container by 0.04 (95% CI: −0.08, −0.01; p = 0.024). Each degree centigrade rise in mean temperature was associated with a decrease in the mean *Aede*s number by 6.49 (95% CI −12.40, −0.58; p = 0.032). Relative humidity (Fig 2C) in previous 30 days of survey had a significant positive impact on *Aedes* abundance, as the mean number of mosquitoes increased by the rise of relative humidity (β = 2.73; 95% CI 0.59, 4.88; p = 0.014).

## Discussion

The present study investigated the abundance and oviposition habitats of *Aedes* mosquitoes in Dhaka during three consecutive monsoon seasons (2011, 2012, and 2013) and one dry season (2012). A broad spectrum of container types within high, medium and low urban socio-economic zones were inspected and *Aedes* larvae and pupae were identified and recorded from several key container types.

Traditionally, *Aedes* abundance is determined by the search for immatures within and around households and these data are used to calculate *Stegomyia* indices such as, HI, BI and CI [27]. These indices not only measure the success of vector control strategy but also help understanding the vector ecology. However, in the contemporary literature, these indices are considered poor methods for predicting vector abundance because of their failure to effectively associate with and explain abundance of adult female mosquitoes and the potential for dengue virus transmission. The determination of dengue risk by interpreting such indices has been questioned by numerous analysts [27,28]. In this regard, Focks (2003) suggested that PI and identifying key and effective containers could be better predictors for disease transmission [27,29]. In the present study, BI, CI, and HI were observed to be lower in 2013 than in 2011 and they remained high in the LSESZ relative to other urban zones. In contrast, PI, did not appear to have any changing pattern during in the four surveys. It remained high in the HSESZ compared to other zones, indicating the “better-offs” in Dhaka are potentially more exposed to dengue risk. The prevailing local proverb that “dengue is a disease of the rich” was supported by the findings of the present study [30]. Although the indices recorded in our investigation were higher than the indices recorded during the dengue outbreak of 2000, the number of reported dengue cases during the study period (2011–2013) was low [17], which can be due to under-reporting and passive nature of the surveillance for human cases of dengue virus infection.

More than one third of Wet Containers (WCs) harboured *Aedes* immatures during the first two wet seasons (2011, 2012). However, fewer WCs were positive in the third survey (2012 monsoon). The ratio of PCs to WCs was observed to be low during the 2012 dry season. Reduced mosquito abundance during the dry season might be due to the effects of lower environmental temperature, very low rainfall, lower relative humidity, and fewer residents storing water during the winter (dry) months. Gradual decreases of the proportion of PCs observed over the monsoon survey periods might be attributed to increased study participant’s awareness regarding dengue vector mosquitoes and their tendency to develop within selected containers within and around HH. Significant numbers of outdoor containers were reported to be positive with immature *Aedes* in the previous studies conducted in urban areas [17,31–33]. As urban dwellers (and the participants in our study) become more aware of the possible oviposition sites of *Aedes* mosquitoes, they apt first to check and clean indoor containers located inside their HHs. Consequently, the vectors tend to shift to the outdoor container, which was clearly observed in the present study. This finding is consistent with several previous studies [17,34–36]. Such adaptive behavior of *Aedes* mosquitoes poses serious challenge to the vector control efforts [37].

Different types of WCs produced variable numbers of *Aedes* immatures during the present study. In all four surveys, plastic drums, plastic buckets, water tanks, clay pots, and flower tubs were observed to producing large number of *Aedes* larvae and pupae. City dwellers of Dhaka use relatively smaller plastic drums, plastic buckets, and larger water tanks to store water from supplied piped water, as municipal water supply is not reliably available. As reported previously, if the water stored within containers is not emptied weekly, they become ideal oviposition sites for *Aedes* mosquitoes [38]. Residents of Dhaka use flower tubs in and around houses to plant relatively small ornamental trees, and refrigerator trays located underneath refrigerator compartment remain out of sight and unnoticed. Tires and clay pots are usually located in outdoor locations where water remains stagnant, become suitable for *Aedes* oviposition. We observed that during the wet seasons, tires and refrigerator trays were mostly responsible for *Aedes* production. These *Aedes* habitats need to be the focus of vector control efforts. It is worth noting here that, novel methods utilizing outdoor containers like tires to downsize *Aedes* mosquito population are being evaluated in comparable countries [39]. In a recent study in Lahore (Pakistan), Malik et al. [40] also noted a distinct seasonality to vector abundance. Periods of high intensity of vector mosquitoes development (i.e., larval observed in standing water environments) occurred during the wetter periods of the season with large numbers of immatures developing within water tanks, bottles, jugs and pitchers. However, during (or shortly after) substantial rainfall events, the occurrence of larvae was low. The increased vector abundance was correlated with periods when the resident human population was more prone to disease outbreaks. Entomological survey results of our study conform to these patterns of optimal range of climatic factors and human behavioural adjustments to various seasonality (such as storing water during dry and/or hot seasons). Also consist with the outcomes of our study, Banerjee et al. [41] in a study of pupal productivity in Kolkata (India), observed that plastic containers were the most productive habitats and tires were least productive sites for *Aedes* mosquitoes. Arduino [42] in the context of coastal urban Brazil, distinguished between movable and fixed containers, and concluded that only three container categories made greater contribution to risk of dengue transmission: metallic and plastic items among the roving containers, and boats/ships among the fixed containers. The potential influence of mobile vs. fixed containers deserves further research attention in various social and ecological contexts. In addition, some previous reports indicate that the shift of container productivity from one type to another is common. For example, in Malaysia, earthenware jars and miscellaneous containers superseded ant traps, buckets, basins, bowls, and concrete tanks as major *Aedes* production site over time [43–46]. Unlike these studies, previously identified key containers in Dhaka including tires, tanks, drums and flowers pots were observed to be preferred oviposition sites for *Aedes* [17].

In the present study, we found that small plastic reservoirs were most abundant and discarded vehicle and construction materials were most efficient immature habitats. Although wide-mouthed large water storage containers like concrete tanks, wells and drums are well-known key oviposition sites in urban areas [47,48] as well as in rural areas [49], we documented that these larger reservoirs were moderately efficient as *Aedes* mosquitoes habitats. Due to adequacy of potential habitats, *Aedes* abundance remains high in Dhaka, like most tropical cities in the developing world, which in turn put people at risk to arboviral infections [50,51]. While Dhaka is presently expanding very rapidly, the required planning for providing healthy environment to the population in the newer areas is generally absent. As a result, the risk of mosquito-borne viral diseases remains high.

In our study, wet containers located in the LSESZ were significantly more likely to be positive for *Ae*. *aegypti* immatures than wet containers located in other SESZs but the distribution of *Ae*. *albopictus* immatures was similar in all SESZ. The higher number of positive wet containers recorded in the LSESZ might be due to inadequate municipal services, such as water supply provisions and garbage collection methods. In addition, in this socioeconomically “worst-off” urban zone, there is a general lack of awareness among the community members about vector breeding sources [36]. However, as stated above, in terms of PI, it has been consistently high in the HSESZ relative to others, indicating that adult *Aedes* survival rate may be much higher in the HSESZ due to supportive habitat (for both immature and adult mosquitoes) in communities with build-up infrastructure. As reported in our study of 2011 data [21], “mosquitoes are everywhere” [36] in Dhaka, and differences among urban zones by socioeconomic status did in fact have nominal effects upon variations in *Aedes* abundance.

In our study, containers filled with rainwater were significantly more likely to be PCs for either *Ae*. *aegypti* or *Ae*. *albopictus*, this conforms to the findings of some previous studies [52–54]. Rainfall is one of the most important environmental factors influencing the production of *Aedes* larvae. Not surprisingly, more *Ae*. *aegypti* and *Ae*. *albopictus* larvae was collected from containers holding rain water. Unsheltered containers that fill with rain water may serve as excellent larval development sites for *Aedes* mosquitoes. Vegetation near these larval production sites might also affect the abundance of mosquito larvae. Trees and shrubs may indirectly enhance mosquito abundance by providing important sugar feeding resources and resting sites. Vegetation may also promote gravid females to lay eggs in locally available containers. Organic detritus of vegetation may alter the aquatic chemistry of the containers, potentially influencing the attractiveness of such containers to gravid females. Moreover, since larvae feed on microorganisms in the water, the organic components in the containers might promote algal and microbe growth, and hence, support mosquito production [55,56]. This is concordant with the findings of the present study, as larval production of any species had been significantly affected by the presence of vegetation near the containers. Effect of shade on the containers did not differ significantly when variables such as SESZ, place of the container and shelter of the container from rain were adjusted. However, high numbers of larvae in the partially shaded and exposed containers was likely due to random factors or as a consequence of rain water storage in those containers made them more attractive for egg-laying females.

In our study, increase in total rainfall was associated with a reduction in mosquito abundance that might have been related to “flushing” of immature forms, especially from containers exposed to rainfall outdoors. The negative impact of rainfall on production of *Aedes* mosquitoes has been reported in several previous studies [57,58]. Furthermore, when coupled with strong winds, heavy rainfall can limit the dispersal and reproduction of the mosquitoes by reducing flight activity, leading to difficulties in finding mates, hosts and suitable larval development sites [58]. During the monsoon surveys, mean temperature varied between 28 and 30°C. In this connection, we observed that an increase in mean temperature was associated with reduction in the abundance of *Aedes* immatures. Warmer temperatures (20–35°C) generally increase the rate of development of dengue vectors, improve or enhance feeding frequency of adult mosquitoes and the rate of dengue virus replication in infected females, so it was surprising that warmer temperatures led to fewer immature Aedes mosquitoes. However, several studies have revealed lag times [59–61] for these relationships. Further investigations are therefore required to determine more precisely how temperature impacts *Aedes* abundance in the Bangladesh context. We observed a positive effect of relative humidity on the abundance of vector mosquitoes in the range between 77–82% relative humidity. It was assumed by previous studies that a higher relative humidity is associated with an increase in hatching of *Aedes* eggs leading to the increased abundance of immature mosquitoes; findings of our investigation conform to this [62,63].

## Conclusion

The present study investigated and revealed the degree and dynamics of the abundance of *Aedes* mosquitoes in different urban SESZs in Dhaka and identified the key containers responsible for harbouring *Aedes* immatures. These containers are chiefly generated and/or possessed by the city dwellers and produce sizeable populations of adult female mosquitoes which increases the risk of pathogen transmission. Proper use, disposal and recycling of the containers by the city dwellers and responsible authorities are necessary for environmental and mosquito habitat management. Community education regarding the biology of *Aedes* mosquitoes, the need to clean HH premises including inverting/discarding unused containers in appropriate ways is anticipated to increase the uptake of these simple but effective intervention strategies. Because the traditional vector indices are not reliable predictors of dengue virus outbreaks, annual mosquito surveillance is necessary to gain a better understanding of how vector populations change over time and how this relates to risk of pathogens that are transmitted by *Aedes* mosquitoes in Bangladesh. Surveillance along with sound prevention and source reduction programs implemented by and within at-risk communities are seen as crucial elements for reducing the risk of dengue and other arboviral infections.

## Limitations

The study was subject to multiple limitations. Firstly, because of accessibility issues, we did not survey containers located on rooftops or potential cryptic larval developmental sites, which may have been important sources of *Aedes* mosquitoes. Secondly, due to a very high rate of change of residence/relocation among Dhaka city dwellers, we could not enrol some HHs in the subsequent surveys. Thirdly, the study collected only immature (larvae and pupae) mosquitoes and not adults, which, if available, may have provided additional valuable information about population structure of the resident vector species. Use of alternative collection methods, such as lethal oviposition traps may have also helped to provide a more robust picture of the vector population structure in this study. Fourthly, species determinations were performed only on a subset of the immature mosquitoes collected in this study; however, the sub-sampling was performed randomly across the different surveys and as such as likely representative of the overall population of mosquitoes encountered. Finally, we did not perform any time series analysis to explore association between climatic factors and mean number of *Aedes*, so the observed association might be true for limited ranges of climatic variables.

## Supporting information

S1 Table Contribution of positive and key premises in the production of *Aedes* positive containers and immature mosquito populations in Dhaka, Bangladesh (2011–2013). (DOCX)

## Abbreviations

BI: Breteau index
CI: container index
HH: household
HI: house index
PC: positive container
PI: pupal index
SESZ: socio-economic status zones
WC: wet container
