# Supplementary material for: Risk factors for the presence of dengue vector mosquitoes, and determinants of their prevalence and larval site selection in Dhaka, Bangladesh
Source: PLoS One. 2018 Jun 21;13(6):e0199457. doi: 10.1371/journal.pone.0199457 (PMC6013170; doi:10.1371/journal.pone.0199457)
Supplement: S1 Table — (DOCX) [file pone.0199457.s001.docx]

**S1 Table.** Contribution of positive and key premises in the production of *Aedes* positive containers and immature mosquito populations in Dhaka, Bangladesh (2011 - 2013)

| Vector survey | Houses inspected | House status | No. of houses (%) | No. of PCs (%) | Mean no./house | | |
| --- | --- | --- | --- | --- | --- | --- | --- |
|  |  |  |  |  | Immature | Larvae | Pupae |
| 2011 Monsoon (Wet) | 884 | Positive premises | 220 | 492 | 19.2 | 16.7 | 2.5 |
|  |  | Key premises | 81 (37) | 308 (63) | 26.8 | 23.2 | 3.6 |
| 2012 Pre-monsoon (Dry) | 546 | Positive premises | 39 | 53 | 13.2 | 10.8 | 2.3 |
|  |  | Key premises | 6 (15) | 11 (21) | 17.7 | 15.7 | 2.0 |
| 2012 Monsoon (Wet) | 899 | Positive premises | 220 | 256 | 25.0 | 18.8 | 6.2 |
|  |  | Key premises | 21 (10) | 49 (19) | 54.3 | 39.5 | 14.8 |
| 2013 Monsoon (Wet) | 639 | Positive premises | 106 | 144 | 22.6 | 18.0 | 4.6 |
|  |  | Key premises | 23 (22) | 48 (33) | 36.2 | 29.8 | 6.3 |
| Total | 2,193 | Positive premises | 534 | 945 | 12,623 | 10,135 | 2,488 |
|  |  | Key premises | 97 (18) | 416 (44) | 4,247 | 3,486 | 761 |
